# RESPONSE AND RETENTION OF AN ONLINE RESEARCH REGISTRY: ADAPTATIONS MADE FOR RECRUITING ASIAN AMERICANS

**DOI:** 10.1093/geroni/igad104.1458

**Published:** 2023-12-21

**Authors:** Kexin Yu, Sarah Gothard, Emily Tupper, Ona Golonka, Allison Lindauer, Aimee Pierce

**Affiliations:** Oregon Health & Science University, Portland, Oregon, United States; Oregon Health & Science University, Portland, Oregon, United States; Oregon Health & Science University, Portland, Oregon, United States; Oregon Health & Science University, Portland, Oregon, United States; Oregon Health & Science University, Portland, Oregon, United States; Oregon Health & Science University, Portland, Oregon, United States

## Abstract

Challenges in recruiting research volunteers, especially racial/ethnic minority older adults, have been documented as one of the most significant obstacles to developing effective treatments for Alzheimer’s Disease and Related Dementia (ADRD). To address the need for recruitment and raise awareness about ADRD research, the Layton Aging and Alzheimer’s Disease Research Center built the online dementia research registry, Alzheimer’s Comprehensive Treatment Network of Oregon and Washington (ACTNOW). Individuals aged 18 and above are eligible to join, and prospective participants self-initiated enrollment into the registry. As of March 2023, there are 497 participants (mean age 63.9, 71.6% female, 74% had bachelor’s or above degree, 12% residing in a rural area) registered in ACTNOW, with an average follow-up period of 1.8 years (SD=1.2, Max=5). To date, 20 research projects have used ACTNOW for their recruitment, and 93% of registry volunteers have been referred to at least one requesting study. The current ACTNOW participants are predominantly non-Hispanic White (92.4%). We will describe ACTNOW’s infrastructure expansion to improve the registry’s capacity for engaging and recruiting older Asian American adults with limited English proficiency. We tested the feasibility of using translated informed consent forms and questionnaires, reconciling data collected in different languages with existing data structures, and the effectiveness of community outreach sessions. Developing the capacity to recruit underrepresented populations for future ADRD research projects can be critical to addressing health disparities in dementia awareness, prevention, diagnosis, and care. Standardizing communications with all registry stakeholders, both researchers and volunteers, helps engagement and accurate recruitment efforts.

